# Animal Magnetism: Metaphoric Cues Alter Perceptions of Romantic Partners and Relationships

**DOI:** 10.1371/journal.pone.0155943

**Published:** 2016-05-26

**Authors:** Andrew G. Christy, Kelly A. Hirsch, Rebecca J. Schlegel

**Affiliations:** Department of Psychology, Texas A&M University, College Station, Texas, United States of America; The University of Nottingham, UNITED KINGDOM

## Abstract

The psychological state of love is difficult to define, and we often rely on metaphors to communicate about this state and its constituent experiences. Commonly, these metaphors liken love to a physical force—it sweeps us off our feet, causes sparks to fly, and ignites flames of passion. Even the use of “attraction” to refer to romantic interest, commonplace in both popular and scholarly discourse, implies a force propelling two objects together. The present research examined the effects of exposing participants to a physical force (magnetism) on subsequent judgments of romantic outcomes. Across two studies, participants exposed to magnets reported greater levels of satisfaction, attraction, intimacy, and commitment.

## Introduction

“Like a river flows

Surely to the sea

*Darling*, *so it goes*

*Some things were meant to be*.*”*

  *-Elvis Presley*, *Can’t Help Falling In Love*

In his hit song “Can’t Help Falling In Love,” Elvis Presley describes the irresistibility of love. The love Elvis sings about is an overwhelming force, motivating him to defy the counsel of “wise men” and “rush in.” Recent empirical work on conceptual metaphor suggests that conceiving of love as a physical force may actually affect the *experience* of love. Specifically, research suggests that when the concrete concept in a metaphor is activated, it can alter perceptions and behaviors related to the abstract concept being represented in that metaphor (i.e., metaphoric transfer; [1, 2]). The current studies thus examine whether exposure to a physical force (magnetism) influences the experience of love (i.e. relationship satisfaction, commitment, intimacy and attraction).

### Metaphoric Representation

According to linguistic perspectives [3, 4 5], metaphors are more than merely aesthetic figures of speech. Rather, metaphors facilitate our understanding of abstract ideas by linking them to concrete concepts grounded in perceptual experience [6, 7, 8, 9]. Further, this metaphoric representation has downstream consequences. According to conceptual metaphor theory [1, 4, 5], activating the concrete concept in a metaphor should alter perceptions and judgments related to the linked abstract concept. Consistent with this idea, Schubert [9] examined the metaphorical representation of power as physical verticality and found that participants judged groups as more powerful when they were presented near the top of a computer screen. Similar patterns have been observed for God-related words presented higher on a screen [6] and for verticality and affect (e.g., “up is good” [7]) as well as other metaphorical links, such as brightness and valence (e.g., “bright is good,” “dark is bad” [8]).

Metaphoric transfer effects have been documented in a variety of domains, including interpersonal outcomes. For example, Williams and Bargh [10] activated the concept of either spatial distance or spatial closeness by asking participants to plot points on a Cartesian plane. Participants primed with distance reported weaker attachments to family members and their hometown relative to participants primed with closeness. Fay and Maner [11] demonstrated that warmth cues increased self-reported desire for social affiliation (see also [12]), but found this effect to be moderated by participants’ levels of attachment avoidance and anxiety. Finally, Landau and colleagues [2] report evidence that activating metaphoric representations of the true-self concept can promote positive intrapersonal outcomes. Specifically, participants primed with the image of an expanding entity (a concrete analog of the true self) reported being more self-actualized and showed less conformity. Taken together, these studies suggest that metaphors impact perceptions of the self and others and thus play an important role in social cognition.

### “Love is a Physical Force”

The current research examines the metaphor “love is a physical force” [5, 13]. This metaphor is instantiated in utterances such as, “We were immediately *attracted* to each other,” and “There was a *magnetism* between us” [5], as well as in the maxim that “opposites attract.” Given the prevalence of these metaphors in ordinary language, it is reasonable to suspect that there are chronically available cognitive associations between the concepts of love and physical force. According to conceptual metaphor theory, in such cases of metaphoric representation the abstract domain (love) is mapped onto the concrete domain (physical force), such that some of the properties of the concrete concept are transferred to the abstract concept [1]. In this case, the concept of love should take on some of the properties of a physical force.

While the previously cited research [10, 11, 12] establishes that metaphoric transfer effects can influence general interpersonal perceptions, other recent work has documented similar effects specific to the romantic domain. For example, Lee and Schwarz [14] found that metaphorically framing love as a unity between two matched parts vs. a mutually-undertaken journey changed how participants responded to conflicts in their relationships. Specifically, being reminded of conflicts had a greater negative impact on relationship satisfaction under a unity frame than under a journey frame. Another recent investigation by Ren, Tan, Arriaga, and Chan [15] found that participants evaluated hypothetical relationship partners (but not their actual partners) more favorably after being exposed to sweet tastes, consistent with the frequent use of sweetness metaphors in pet names (e.g. *sweetheart*). These findings show that romantic outcomes can be impacted by exposure to metaphoric cues.

The present studies examined the impact of a previously-unexplored metaphor for romantic attraction, namely the physical force of magnetism. In a novel manipulation, participants were randomly assigned to handle either magnetized or non-magnetized plastic blocks. Critically, this particular metaphor is one in which the concrete source concept (i.e. magnetism) is *experientially remote* from the abstract target concept (i.e. love; [1]). This means that there is relatively little overlap between experiences of magnetism and experiences of romantic love. In contrast, other interpersonally-relevant metaphors, such as “intimacy is closeness” [10] and “kindness is warmth” [11, 12], are cases where the concrete source concepts actually overlap in experience with their corresponding abstract target concepts. In early life, our experiences of emotional intimacy and kindness reliably co-occur with experiences of proximity and physical warmth. In these cases, embodied-cognitive mechanisms may be invoked to explain the effects of metaphoric manipulations (i.e. manipulations of the source concept produce bodily states similar to the bodily states associated with the target concept). In cases where the target and source concepts are experientially remote, such as our magnetism manipulation, or Ren et al.’s sweetness manipulations [15], embodied-cognitive mechanisms are not viable explanations. Rather, the effects of these manipulations must primarily be explained as a function of cognitive associations between the target and source concepts that have been established by the habitual use of linguistic metaphors. This is a key distinction between conceptual metaphor theory and theories of embodied cognition; the latter do not predict effects in cases where the source concept is experientially remote from the target concept.

To the best of our knowledge, only one prior study has examined physical-force metaphors for love in any way. Allbritton, McKoon, and Gerrig [16] presented participants with passages that used an electricity metaphor in the initial sentence (“*The electricity between John and Martha was overwhelming*.”) and final sentence (“*Suddenly*, *sparks were flying*.”). In a metaphor-consistent condition, the intervening text described a passionate encounter between John and Martha. In a metaphor-inconsistent condition, the intervening text described John knocking a lamp into a punchbowl, providing a literal source for the flying sparks. Recognition of the final sentence was faster among participants who received the metaphor-consistent passage, indicating that metaphor-consistent passages were processed more efficiently. This further supports the suggestion that physical-force analogies for love are chronically available and may be an important part of people’s schemas for love and attraction. The current research differs from this study by investigating whether physical-force cues influence perceptions of one’s own relationship in metaphor-consistent ways.

### Overview of the Current Studies

The current studies were designed to investigate whether activating a metaphorical representation of “love as a physical force” influenced the experience of love (i.e., attraction, intimacy, commitment, and satisfaction). Based on conceptual metaphor theory, we predicted that people who experienced a physical force would report greater “love” than people who did not. In order to manipulate the experience of a physical force, participants brought together two blocks that were either magnetized (attraction condition) or nonmagnetized (control condition) for approximately one minute. Following this task, participants completed the dependent measures assessing their perceptions of their own relationships.

## Study 1

### Methods

#### Participants

One hundred and twenty (80 female) students enrolled in an introductory psychology course at Texas A&M University participated in Study 1 for partial fulfillment of a course requirement. Ages ranged from 18 to 22 (*M* = 18.82, *SD* = .85).

Students were eligible to participate in the study only if they had reported being in a romantic relationship on a pre-screening questionnaire at the beginning of the semester. Ninety-three participants (78.3%) reported still being in a relationship at the time of data collection. Participants who were no longer in relationships at the time of the study were asked to think of their most recent relationship partner as they completed the dependent measures. Analyses comparing the results in the complete sample vs. only those participants currently in relationships revealed that although some *p*-values differed somewhat across the two samples, the pattern of means was always the same. These results are available in the supplementary materials. All results in this report are for the complete sample.

Represented races included 74.1% European American, 11.7% Hispanic/Latino, 4.2% Asian, 4.2% Black, 4.2% multiracial, .8% American Indian, and .8% Hawaiian/Pacific Islander.

#### Materials and procedure

All materials and procedures used in the present studies were approved by the Texas A&M University IRB. Upon arrival to the lab, participants were escorted to private rooms, each containing a single computer, and were informed that they would be participating in a study about relationships and the self. Written consent was not obtained from participants, in order to eliminate records linking participants’ identities to the study. Consent was obtained by having participants read an information sheet about the study, which was the first screen of the study visible when participants were seated at their computer stations. Participants indicated their consent to be in the study by clicking “next” at the bottom of this information sheet; a procedure approved by the Texas A&M IRB. Participants were randomly assigned to one of three conditions: attraction, control, and repel.

*Attraction manipulation*: Participants were presented with a set of two blocks that were either magnetized such that the blocks were attracted to each other (attraction condition), were non-magnetized (control condition), or were configured to repel one another (repel condition). In all conditions, participants were given the following instructions:

*We all know that college students have busy and sometimes stressful lives*. *Studies have shown that doing a simple physical activity as a “mental break” before engaging in a task that requires mental effort reduces stress and aids in concentration and performance*.

*On the next page*, *you will be presented with instructions on how to do one of the simple physical tasks used in these prior studies*. *Following the activity*, *you will be presented with a series of questionnaires*.

**Fig 1:**
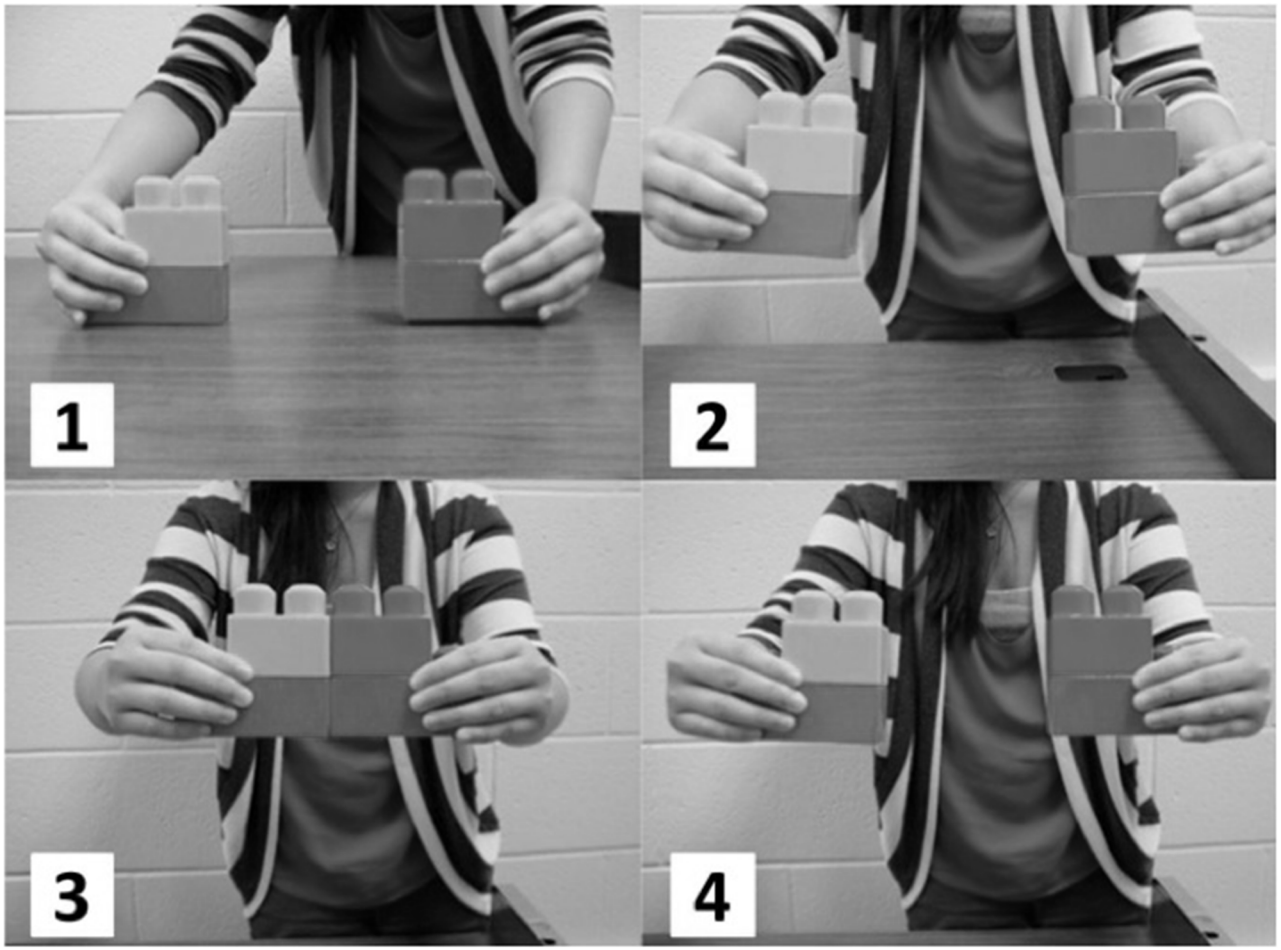
Instructions for block task. Please pick up the blocks in front of you and bring them together as shown in the pictorial instructions below. Plese do this repeatedly until the program advances automatically (in 1 minute).

Before beginning the block task, participants received pictorial directions (see Fig 1) instructing them to bring the blocks together repeatedly for one minute. The computer program did not advance until one minute had elapsed. Participants were not observed directly during the study, so no data on their compliance with the instructions is available. While this is a limitation of the present studies, we have no reason to suspect that any substantial proportion of the participants in either study was non-compliant.

*Perceptions of romantic relationships*: Participants then completed the dependent measures. All measures made use of the same 7-point response scale (1 = strongly disagree; 7 = strongly agree). Dependent measures included four relationship-relevant perceptions: global relationship satisfaction ([17]; *M* = 5.75, *SD* = 1.14, *α* = .90; e.g. “I feel satisfied with our relationship”); intimacy (Emotional Intimacy Scale [18]; *M* = 6.19, *SD* = .94, *α* = .88; “I can openly share my deepest thoughts and feelings with my partner”), attraction ([19]; *M* = 6.02, *SD* = 1.00, *α* = .84; e.g. “I feel a great deal of sexual desire for my partner”) and commitment ([17]; *M* = 5.84, *SD* = 1.27, *α* = .93; e.g. “I want our relationship to last forever”).

*Additional measures*: After completing the primary dependent measures, participants completed a number of other measures that were included for exploratory purposes, which will not be discussed further in this report. These included the Implicit Theories of Relationships Scale [20], the brief version of the Personal Meaning Profile [21], the short form of the Experiences in Close Relationships Scale [22], the Rosenberg self-esteem scale [23], and a 24-item valence-arousal mood measure based on the adjectives provided by Tsai [24]. The order in which these measures have been listed reflects the order in which they were presented to participants. After completing these measures, participants completed demographic items and items probing for suspicion and distraction, prior to being debriefed.

### Results

#### Preliminary analysis

To test the hypothesis that attraction cues (i.e. magnetized blocks) influence the experience of “love”, a multivariate ANOVA was conducted with condition (attraction vs. control vs. repel) entered as a fixed factor and relationship satisfaction, intimacy, attraction, and commitment entered as outcome variables. Detailed results for this analysis can be found in the supplementary materials (S1 File). In general, results of this analysis indicated that the various indicators of relationship quality were elevated in the attraction condition relative to the repel and non-magnetized control conditions, while the latter two conditions did not differ significantly (all *p*s = 1.00 for Bonferroni-adjusted pairwise comparisons).

#### Primary analysis

Given the lack of differences observed between the repel and control conditions in the preliminary analysis, we combined these conditions in the primary analysis. Subsequently, we refer to these conditions as the attraction (*n* = 41) and combined control (*n* = 79) conditions.

We conducted an independent-samples *t*-test comparing the attraction and combined control conditions. Levene’s test indicated that the assumption of equal variances was violated for these relationship satisfaction, attraction, and commitment, and accordingly the results for these variables are reported with adjusted degrees of freedom. Results of this analysis indicated that there were significant differences between the conditions on three out of the four dependent variables, namely relationship satisfaction, *t*(109.24) = 2.85, *p* = .005, attraction, *t*(114.05) = 2.26, *p* = .026, and commitment, *t*(114.58) = 2.91, *p* = .004. No significant differences were found on the emotional intimacy variable, *t*(118) = 1.22, *p* = .226. Means, effect sizes, and 95% confidence intervals for these comparisons can be found in Table 1.

**Table 1:** Means, standard deviations, effect sizes, and confidence intervals in Studies 1 & 2.

Variables	Mean (Standard Deviation)		Effect Size (Cohen’s *d*)	95% Confidence Interval of the Difference
	Attraction Condition	Control Condition		
*Study 1*	6.11 (.84)	5.56 (1.23)	.52	.17 –.92
Relationship Satisfaction				
Emotional Intimacy	6.34 (.82)	6.12 (1.00)	.24	-.14 –.58
Attraction	6.26 (.69)	5.89 (1.11)	.40	.05 –.70
Commitment	6.24 (.86)	5.63 (1.40)	.53	.19–1.01
*Study 2*	5.29 (1.38)	4.83 (1.33)	.34	.02 –.90
Relationship Satisfaction				
Emotional Intimacy	5.82 (1.28)	5.37 (1.33)	.34	.03 –.87
Attraction	5.69 (1.23)	5.08 (1.37)	.47	.18–1.02
Commitment	5.26 (1.61)	4.81 (1.45)	.29	-.04–.95
Romance Accessibility	1.68 (.96)	1.36 (.85)	.35	.03 –.61

## Study 2

While the results of Study 1 are consistent with our hypotheses, the novelty of our manipulation leaves some doubt about the reliability of these results, especially in light of current concerns about the replicability of effects in psychology [25]. Conducting a direct replication, that is, repeating a study using identical materials and procedure, is an important first step in establishing that an effect is robust and not a product of error. The only way in which the manipulation used in Study 2 deviated from that used in Study 1 was the omission of the repel condition from Study 2. We decided to drop this condition from Study 2 because it did not differ in any way from the non-magnetized condition in Study 1 (see supplementary materials). Dropping the repel condition allowed us to more easily conduct a higher-powered replication of the major findings from Study 1.

### Methods

#### Participants

One hundred and fifty (82 female) students enrolled in introductory psychology at Texas A&M University participated in Study 2 for partial fulfillment of course requirements. Ages in this sample ranged from 18 to 48 (*M* = 19.80, *SD* = 3.25). All participants were selected based on their responses to pre-screening questionnaires in which they indicated they were currently in a relationship. Participants’ relationship status was not recorded at the time of participation, due to a programming error. The racial composition of the sample was 73.4% White, 16.0% Hispanic/Latino, 5.3% Asian, 3.3% Black, 1.3% multiracial, and .7% Hawaiian/Pacific Islander. One participant did not report their race.

#### Materials and procedure

Materials and procedure for obtaining consent, delivering the manipulation, and collecting primary dependent measures were identical to those used in Study 1, excepting the omission of the “repel” condition. Descriptive statistics were as follows for the dependent measures: relationship satisfaction (*M* = 5.06, *SD* = 1.37, *α* = .91), intimacy (*M* = 5.60, *SD* = 1.32, *α* = .90), attraction (*M* = 5.38, *SD* = 1.33, *α* = .89), and commitment (*M* = 5.03, *SD* = 1.54, *α* = .93).

Overall, the means for the dependent measures were considerably lower in Study 2 than in Study 1 (we thank an anonymous reviewer at *PLOS ONE* for calling this difference to our attention). Based on the pattern of means in Study 1, we believe that this difference is due to a larger proportion of Study 2 participants no longer being in relationships at the time of the study. Participants in relationships at the time of Study 1 scored significantly higher on all four dependent measures than participants who were no longer in relationships (see supplementary materials for detailed results). Unfortunately, data for relationship status at the time of Study 2 was not recorded due to a computer error, so we are unable to directly assess whether there were actually more participants currently in relationships at the time of Study 2.

*Accessibility of romantic thoughts*: After completing the primary dependent measures, participants then completed a nine-item measure of romantic-thought accessibility (RTA) based on death-thought accessibility measures used in terror management research [26]. This measure was included to assess the possibility that the manipulation functioned to make thoughts of romantic love more cognitively accessible to participants in the attraction condition. Each item in this scale consisted of a word fragment with between one and three letters missing. Five out of the nine fragments could be completed either with words related to or unrelated to romance. The five target word fragments were: KI_ _ (romantic completion: “kiss”), LO_ _ (romantic completion: “love”), C_ _ _LE (romantic completion: “cuddle”), H_G (romantic completion: “hug”) and PASS_ _ _ (romantic completion: “passion”). The number of romantic completions for each participant was summed across these five items (*M* = 1.52, *SD* = .92)

*Additional measures*: Following the primary dependent measures, participants completed the following additional measures, which were included for exploratory purposes and will not be discussed in detail in this report. These measures were all created by the second author. They included the same mood measure used in Study 1 [24], a 25-item measure of explicit endorsement of love metaphors, a measure in which participants rank order six statements representative of Lee’s love styles [27], and finally a measure in which participants are asked to list the five “most important things” in their life. As before, the order in which these measures have been listed is the order in which participants completed them, and after completing these measures participants reported demographic information, were probed for suspicion and distraction, and debriefed.

### Results

#### Primary analysis

As in Study 1, an independent-samples *t*-test was conducted with condition (attraction vs. control) as the grouping variable, and the four relationship variables from Study 1 plus the romantic-thought accessibility measure entered as test variables. Levene’s test indicated no violations of the assumption of equal variances. Results of the *t*-test indicated that there were significant between-groups differences for four of the five variables. Specifically, participants who had interacted with magnetized blocks reported enhanced relationship satisfaction, *t*(148) = 2.07, *p* = .040, emotional intimacy, *t*(148) = 2.10, *p* = .038, attraction, *t*(148) = 2.84, *p* = .005, and made more romance-related completions on the RTA measure, t(148) = 2.16, p = .032. A marginal effect of condition was observed on commitment, *t*(148) = 1.82, *p* = .072, with means falling in the predicted direction. Means, effect sizes, and 95% confidence intervals for these comparisons can be found in Table 1.

#### Exploratory analyses of indirect effects

Since an effect of condition was observed on the RTA measure, we followed up with analyses using the PROCESS macro for SPSS [28] to test for the presence of indirect effects of condition through the accessibility of romantic cognitions on the primary dependent variables. We ran four models (corresponding to Model 4 described by Hayes [28]), one for each of the primary dependent variables (entered as Y variables in the mediation model), in which condition was entered as the independent variable (X) and scores on the RTA measure were entered as the mediating variable (M). Bias-corrected confidence intervals for the indirect effects were estimated based on 5000 bootstrap samples. Results indicated that there were significant indirect effects of condition via romantic-thought accessibility on relationship satisfaction, *b* = -.07, *b*_*SE*_ = .05, 95% CI [-.216, -.002], attraction, *b* = -.07, *b*_*SE*_ = .05, 95% CI [-.228, -.002], and on commitment, *b* = -.09, *b*_*SE*_ = .07, 95% CI [-.284, -.003]. Even though there was not a conventionally significant direct effect of condition on commitment, indirect effects can still occur in the absence of significant direct effects [29, 30]. No indirect effects through RTA were observed for intimacy, *b* = -.02, *b*_*SE*_ = .04, 95% CI [-.123, .061]. The complete PROCESS outputs for each dependent variable are included in S1 File.

These results provide some support for the idea that the observed effects of the experimental manipulation are achieved by activating the concept of romantic love via exposure to the metaphorically-related target concept of magnetism. However, caution is warranted in interpreting these effects given the correlational nature of these mediational analyses [31]. Although alternative models, in which RTA was entered as the outcome variable and relationship satisfaction, attraction, intimacy, and commitment were entered as mediators, were generally not as well-supported as the models reported here (see S1 File), these or other alternative models may nonetheless provide viable accounts of the mechanism underlying the present effects.

## Discussion

In two studies, interacting with magnetized blocks enhanced participants’ perceptions of satisfaction, attraction, and commitment in their romantic relationships. In the second study, this manipulation was also found to enhance perceptions of emotional intimacy. These findings are consistent with the suggestion that love is represented metaphorically as analogous to a physical force. Importantly, the metaphoric connection between love and physical force was never explicitly referenced in the present studies. Indeed, no explicit reference to physical force was ever made; participants’ only exposure to this concept was achieved via exposure to a physical force (i.e. magnetism) under the guise of taking a “mental break”. As such, the present research is a case where a primarily *linguistic* metaphor, one that does not have an apparent basis in physical experience, may be activated by nonlinguistic means [32]. Given that there is relatively little experiential overlap between the target and source concepts in the present research, these effects may be thought of as fairly “pure” metaphoric effects since other potential mechanisms such as embodied cognition are less clearly applicable [1]. In contrast to other metaphors pertaining to interpersonal intimacy, such as warmth [11, 12] and physical proximity [10], experiences of the source concept in the present studies (magnetism) do not co-occur nearly as reliably with experiences of the target concept of romantic attraction. The metaphoric association between magnetic attraction and romantic attraction is based on conceptual similarities between these ideas (i.e. our experience of being irresistibly drawn to a potential lover is similar to the inevitability of magnets’ attraction to one another) rather than on the co-occurrence of the source and target concepts in embodied experience early in life (e.g. the experience of being physically close and warm when being held by one’s parent or other caregiver). This is important because linguistic metaphors have received far less attention in the social-psychological literature than embodied metaphors. The present studies demonstrate that embodied exposure to source concepts can activate linguistic metaphors, even though the mediating processes by which the target concept is influenced are not themselves embodied in these cases.

It is important to note that the present studies provide limited evidence about the mechanism by which the observed effects were achieved. While conceptual metaphor theory suggests that the basic mechanism is similar to spreading activation [1], the present studies provide only some direct evidence that the manipulation activated the concepts of magnetism or attraction (romantic or otherwise). At least two potential mechanisms are consistent with the present findings, and with the general tenets of conceptual metaphor theory. First, it is possible that activation of the concept of magnetism spread to the metaphorically linked concept of romantic attraction, resulting in heightened cognitive accessibility of the latter. After exposure to magnets it may have been easier for participants to retrieve information related to romantic attraction, resulting in inflation of their reported satisfaction, attraction, intimacy, and commitment to their partners. If this is the case, the observed pattern of results is an instance of the availability heuristic [33], albeit one activated by a physical experience and driven by metaphorical associations between concepts. The results for the romantic-thought accessibility (RTA) variable in Study 2 are consistent with this interpretation; scores on this measure were elevated in the attraction condition and analyses using PROCESS provided evidence that RTA mediated the effects of condition on some of the dependent measures, namely attraction and commitment.

A second possibility is that some specific feature(s) of the source concept (magnetism) are mapped onto the target concept (romantic love), resulting in the differences between the experimental and control conditions observed in the present research. That is, exposure to magnetism may actually have changed participants’ experience of romantic attraction in certain ways that led them to report greater satisfaction, intimacy, attraction, and commitment. This mechanism of *metaphoric transfer* [1] is unique to conceptual metaphor theory. For instance, it may be that the deterministic, irresistible nature of magnetic attraction is transferred to the concept of romantic love. If one’s feelings for one’s partner are perceived as irresistible, this may motivate one to report that one’s relationship is better or more ideal [34]. The results for implicit theories of relationships and sources of meaning in Study 1, and for metaphor endorsement in Study 2 (see S1 File) may speak against this interpretation—at the very least the manipulation does not seem to have changed participants’ *explicit* endorsement of various lay theories and metaphoric conceptions of love. However, it is possible that such changes occurred at an implicit level, such that while participants’ explicit beliefs about love were not affected, their underlying intuitions and criteria for evaluating their own relationships did shift in a metaphor-consistent direction.

Future research should attempt to distinguish between these and other potential mechanisms in metaphoric effects. This is not to imply that one of these suggested mechanisms provides the correct account for *all* metaphoric effects. Indeed, it is most likely that some of these effects are driven by availability as a consequence of spreading activation, while others are driven by more specific metaphoric transfer effects, and others (perhaps most) are produced by the simultaneous operation of both processes. Parsing the exact role of each mechanism would be an important advancement in our understanding of conceptual metaphors, and as such is a fruitful area for future investigation.

Since the present studies do not unequivocally demonstrate the mechanism underlying the effects, it is worth considering alternative explanations not derived from conceptual metaphor theory. For example, the tasks may have differed systematically on dimensions other than metaphoric activation, such as ease/difficulty (we are grateful to an anonymous reviewer at *PLOS ONE* for bringing this issue to our attention). That is, it may have been easier for participants to perform the task in the attraction conditions relative to the control conditions, since the magnetized blocks in a sense complete the task *for* the participants. Although we do not have direct data assessing participants’ experience of ease, this seems unlikely given that the magnets makes it easier to bring the blocks together but also more difficult to subsequently separate. However, given that the text of the instructions stated that the goal of the block task was to bring the blocks *together*, we cannot altogether rule out the possibility that the ease of this goal-relevant action in the attraction conditions was driving the observed effects. Another, perhaps more plausible possibility is that the task was more enjoyable or interesting in the magnetized conditions relative to the non-magnetized condition. While there were some hints in some exploratory mood analyses that this may be the case (see supplementary materials), these effects were neither consistent nor conclusive. Further, on this view the attraction and repel conditions should resemble one another, assuming that any task in which the blocks are magnetized is more interesting/enjoyable than the non-magnetized block task. However, this was not the case (see supplementary materials for detailed results). Thus, while this alternative explanation cannot be completely ruled out, a metaphor-based explanation seems best suited to account for the dissimilarity of the attraction and repel conditions. In general, the lack of effects on variables other than the primary dependent measures speaks to the specificity of the present effects, which is most consistent with a metaphoric account.

The failure of the repel condition in Study 1 merits further consideration, as it may be informative about the nature of magnetism metaphors for romantic love. One compelling possibility is that while there is a strong metaphoric connection between magnetic attraction and interpersonal attraction, the connection between magnetic repulsion and interpersonal repulsion is much weaker. This is plausible on multiple grounds. First, attraction seems to play a much more central and defining role in the folk concepts of magnets and magnetism than repulsion does. People most often use magnets for their properties of attraction, and the set of objects that a magnet will be attracted to (i.e. all ferrous metals) is considerably larger than the set of objects that a magnet will repulse (i.e. the opposing poles of other magnets). Thus, metaphoric representations involving magnetism will most likely prioritize attraction over repulsion. Further, there may be other source concepts for interpersonal repulsion that are much more salient than magnetic repulsion. For example, people may rely on embodied experiences of nausea or disgust towards noxious stimuli as their primary source concepts for understanding interpersonal repulsion.

Even assuming that magnetic repulsion is an important metaphoric source concept for interpersonal repulsion, the dependent measures (i.e. measures of positive relationship perceptions) and the targets evaluated (i.e. romantic partners) in the current studies may not have been well-suited to capturing the effects of the repel condition. Had we included a measure of disgust towards one’s partner in Study 1, we may have observed elevated scores on such a measure in the repel condition. Alternatively, it may be that participants felt so little disgust or repulsion towards their partners to begin with that these feelings remained at a trivial level even when amplified by exposure to the metaphoric cue of magnetic repulsion. If this were the case, it would be necessary to have participants evaluate targets toward whom they harbor at least some pre-existing disgust or repulsion in order to observe effects of the repel condition. Ultimately, further empirical work is needed to conclusively account for why the repel condition failed in the current investigation.

While further work is needed to completely understand the mechanisms underlying the present effects and the nature of magnetism metaphors, our results clearly indicate that exposure to magnetism can function to promote romantic attraction. Further, the results for the romantic-thought accessibility measure in Study 2 provide some evidence that magnets produce these effects by making romantic cognitions more salient. This adds to a growing body of research demonstrating the interpersonal consequences of exposure to various metaphoric cues, including distance [10], warmth [11, 12], sweet tastes [15] and unity vs. journey framings [14]. Conceptually, our magnetism manipulation is most similar to the sweet-taste manipulations used by Ren et al. [15]. As mentioned previously, both of these tasks involve metaphors in which the source and target concepts are experientially remote, meaning that the association between these concepts is primarily linguistic rather than being rooted in the co-occurrence of the source and target concepts in lived experience. However, where Ren et al. found that their manipulation influenced perceptions of hypothetical, but not actual, romantic partners, we found that our manipulation changed how participants felt about their actual partners, both current and past. As such, this task may be of use to researchers who are seeking to manipulate participants’ degree of attraction to their actual partners.

The current studies speak to the pervasive influence of linguistic conventions on our conceptual systems, indicating that idiomatic ways of referring to certain objects and events can guide perceptions and judgments when those objects and events are encountered in life. The present research suggests that such influences can occur even when the objects in question are highly familiar, such as romantic partners, and even when the metaphors are primarily linguistic, involving target concepts that are experientially remote from the corresponding embodied source concepts. As such, these results drive home the basic premise of conceptual metaphor theory—that metaphors are not mere linguistic ornaments, but that they constrain and influence cognition in meaningful ways.

## Supporting Information

S1 File Supplementary Analyses & Results. Detailed reports of results not included in the main document.
(PDF)

S2 File Study 1 Data—SPSS. Study 1 data in SPSS (.sav) file format.
(SAV)

S3 File Study 1 Data—CSV. Study 1 data in comma-delimited (.csv) file format.
(CSV)

S4 File Study 2 Data—SPSS. Study 2 data in SPSS (.sav) file format.
(SAV)

S5 File Study 2 Data—CSV. Study 2 data in comma-delimited (.csv) file format.
(CSV)
