# Supplementary material for: Animal Magnetism: Metaphoric Cues Alter Perceptions of Romantic Partners and Relationships
Source: PLoS One. 2016 May 26;11(5):e0155943. doi: 10.1371/journal.pone.0155943 (PMC4881897; doi:10.1371/journal.pone.0155943)
Supplement: S1 File — Detailed reports of results not included in the main document. (PDF) [file pone.0155943.s001.pdf]

Supplementary Materials

MANOVA Results for Primary DVs in Study 1

We conducted a multivariate ANOVA with condition (attraction vs. repel vs. non-magnetic) as a fixed factor and relationship satisfaction, attraction, intimacy, and commitment as dependent variables. Significant omnibus effects of condition were observed for relationship satisfaction, $F(2, 117) = 3.22$, $p = .043$, partial $\eta^2 = .05$, and commitment, $F(2, 117) = 3.85$, $p = .024$, partial $\eta^2 = .05$. No significant effects were observed for attraction, $F(2, 117) = 1.92$, $p = .151$, or intimacy, $F(2, 117) = .84$, $p = .434$.

Post-Hoc Tests: Bonferroni-Adjusted

Bonferroni-adjusted post-hoc analyses indicated that relationship satisfaction was marginally higher in the attraction condition ($M = 6.11$) than in the control condition ($M = 5.54$), $p = .070$, 95% CI_{diff} [-.03, 1.18], and trending towards being higher than the repel condition ($M = 5.59$), $p = .123$, 95% CI_{diff} [-.09, 1.27]. Relationship satisfaction did not differ between the control and repel conditions, $p = 1.00$, 95% CI_{diff} [-.67, .56]. Similarly, commitment was also marginally higher in the attraction condition ($M = 6.24$) relative to both the control condition ($M = 5.62$), $p = .085$, 95% CI_{diff} [-.06, 1.29], trending towards being higher than the repel condition ($M = 5.65$), $p = .111$, 95% CI_{diff} [-.09, 1.27], and did not differ between the control and repel conditions, $p = 1.00$, 95% CI_{diff} [-.66, .71]. With respect to romantic attraction, the attraction condition ($M = 6.26$) was non-significantly higher than the control condition ($M = 5.87$), $p = .235$, 95% CI_{diff} [-.14, .93] and the repel condition ($M = 5.91$), $p = .339$, 95% CI_{diff} [-.19, .90]. Finally, emotional intimacy did not differ between the attraction ($M = 6.34$) and repel conditions ($M = 6.07$), $p = .611$, 95% CI_{diff} [-.24, .78], between the attraction and control conditions ($M = 6.17$), $p = 1.00$, 95% CI_{diff} [-.34, .68], or between the repel and control conditions, $p = 1.00$, 95% CI_{diff} [-.61, .42].

Post-Hoc Tests: LSD/No Adjustments

LSD post-hoc analyses (i.e. with no adjustments for multiple comparisons) indicated that relationship satisfaction in the attraction condition was significantly higher than in the control condition, $p = .023$, 95% CI_{diff} [.08, 1.07], and the repel condition, $p = .041$, 95% CI_{diff} [.02, 1.01]. Relationship satisfaction did not differ between the control and repel conditions, $p = .828$, 95% CI_{diff} [-.45, .55]. Commitment was also higher in the attraction condition than in the control condition, $p = .015$, 95% CI_{diff} [.13, 1.21] and the repel condition, $p = .022$, 95% CI_{diff} [.09, 1.18], and did not differ between the repel and control conditions, $p = .903$, 95% CI_{diff} [-.58, .51]. Romantic attraction in the attraction condition was marginally higher than in the control condition, $p = .078$, 95% CI_{diff} [-.05, .83], and trending towards being higher than the repel condition, $p = .113$, 95% CI_{diff} [-.09, .80]. Romantic attraction did not differ between the control and repel conditions, $p = .867$, 95% CI_{diff} [-.48, .41]. Finally, emotional intimacy did not differ between the attraction and control conditions, $p = .415$, 95% CI_{diff} [-.24, .59], between the attraction and repel conditions, $p = .204$, 95% CI_{diff} [-.15, .69], or between the repel and control conditions, $p = .644$, 95% CI_{diff} [-.32, .52].

Overall, these patterns of results are consistent with our predictions – indicators of relationship quality were consistently elevated in the attraction condition relative to the other two conditions, and no differences approaching significance were observed between the repel and control

conditions. As such, in the primary analysis reported in the main text we collapsed across the repel and control conditions.

Results for Participants in Relationships at the time of Study 1

We conducted an independent-samples *t*-test with relationship status (currently in vs. not in a relationship) as the test variable and relationship satisfaction, commitment, attraction, and intimacy entered as dependent variables. Levene's test indicated that the variances between the two groups were unequal, hence the subsequent results are reported with adjusted degrees of freedom. Self-reported attraction was higher among participants currently in relationships (M = 6.19) than those not in relationships (M = 5.43), $t(32.05) = 2.82$, $p = .008$. Intimacy was also higher among those in relationships (M = 6.36) than those not in relationships (M = 5.61), $t(31.55) = 2.91$, $p = .007$. Similarly, participants in relationships reported greater relationship satisfaction (M = 5.94) than those not in relationships (M = 5.10), $t(34.73) = 2.97$, $p = .005$. Finally, commitment was also higher among participants in relationships (M = 6.08) than those not in relationships (M = 5.02), $t(33.05) = 3.29$, $p = .002$.

These results are unsurprising given that participants who were no longer in relationships at the time of the study were instructed to think about their most recent relationship partners when completing the dependent measures. However, even participants no longer in relationships tended to offer positive evaluations of their former relationships and partners.

On the subset of participants currently in relationships (n = 93), we next conducted a multivariate ANOVA with condition (attraction vs. repel vs. non-magnetic) as a fixed factor and relationship satisfaction, attraction, intimacy, and commitment as dependent variables. Results of this analysis indicated only a marginal effect on relationship satisfaction, $F(2, 90) = 2.62$, $p = .073$, partial $\eta^2 = .06$. No significant omnibus effects of condition were observed for attraction, $F(2, 90) = .821$, $p = .443$, partial $\eta^2 = .02$; for intimacy $F(2, 90) = 2.07$, $p = .132$, partial $\eta^2 = .04$; or for commitment, $F(2, 90) = 2.03$, $p = .137$, partial $\eta^2 = .04$. Bonferroni-adjusted post-hoc analyses indicated that relationship satisfaction was non-significantly elevated in the attraction condition (M = 6.26) relative to the repel condition (M = 5.74), $p = .125$, and the control condition (M = 5.78), $p = .179$. A similar, but less pronounced, pattern of means was observed for attraction ($M_{\text{attract}} = 6.33$, $M_{\text{repel}} = 6.07$, $M_{\text{control}} = 6.15$), intimacy ($M_{\text{attract}} = 6.53$, $M_{\text{repel}} = 6.15$, $M_{\text{control}} = 6.39$), and commitment ($M_{\text{attract}} = 6.37$, $M_{\text{repel}} = 5.88$, $M_{\text{control}} = 5.96$). None of the pairwise comparisons for these dependent variables approached significance (all *p*s .199 or greater). Although none of the pairwise comparisons between conditions were significant in this analysis, the pattern of means is consistent with predictions. Furthermore, these comparisons may have lacked sufficient power to detect effects in the reduced sample.

We also conducted an independent-samples *t*-test with the collapsed repel and control conditions, mirroring the primary analysis reported in the manuscript, with the subsample of participants currently in relationships. Levene's test indicated that variances were unequal between the attraction condition (n = 33) and the collapsed control condition (n = 60), and accordingly the following results are reported with adjusted degrees of freedom. Relationship satisfaction was higher in the attraction condition (M = 6.25) than in the control condition (M = 5.76), $t(89.27) = 2.64$, $p = .010$, 95% CI_{diff} [.12, .87]. Commitment was also higher in the attraction condition (M = 6.37) than in the control condition (M = 5.92), $t(89.12) = 2.28$, $p = .025$, 95% CI_{diff} [.06, .85].

Intimacy was marginally higher in the attraction condition (*M* = 6.53) than in the control condition (*M* = 6.27), $t(90.94) = 1.90$, $p = .061$, 95% CI_{diff} [-.01, .53]. Attraction did not significantly differ between the attraction condition (*M* = 6.33) and the control condition (*M* = 6.11), $t(85.68) = 1.35$, $p = .180$, 95% CI_{diff} [-.10, .53]. Overall, these results are consistent with predictions and generally mirror those obtained for the full sample, with some minor discrepancies. Specifically, there was an effect on attraction and no effect on intimacy in the full sample, while among participants in relationships there was no effect on attraction, but a marginal effect on intimacy was observed. We do not believe these differences are especially meaningful. They likely reflect the fact that scores on the dependent measures were overall higher and less variable among participants in relationships than in the full sample.

Results for Additional Measures in Studies 1 and 2

Study 1

Mood

We conducted a multivariate ANOVA with condition (attraction vs. repel vs. non-magnetic) as a fixed factor and positive and negative affect entered as dependent variables. Results indicated no effect of condition on positive affect, $F(2, 117) = .41$, $p = .662$, partial $\eta^2 = .007$. A marginal omnibus effect was observed for negative affect, $F(2, 117) = 3.07$, $p = .050$, partial $\eta^2 = .05$. Bonferroni-adjusted post-hoc tests indicated that negative affect was non-significantly elevated in the control condition (*M* = 3.13) relative to both the attraction condition (*M* = 2.66), $p = .119$, and the repel condition (*M* = 2.63), $p = .088$. The attraction and repel conditions did not differ from one another, $p = 1.00$.

Self-Esteem

We conducted an ANOVA with condition (attraction vs. repel vs. non-magnetic) as a fixed factor and self-esteem as the dependent variable. Results indicated no effect of condition on self-esteem, $F(2, 117) = .10$, $p = .907$, partial $\eta^2 = .002$.

Implicit Theories of Relationships

We conducted a multivariate ANOVA with condition (attraction vs. repel vs. non-magnetic) as a fixed factor and the Destiny and Growth subscales of the Implicit Theories of Relationships scale (Knee, Patrick, & Lonsbary, 2003) as dependent variables. Results indicated no effect of condition on either the Destiny subscale, $F(2, 117) = .59$, $p = .558$, partial $\eta^2 = .01$, or on the Growth subscale, $F(2, 117) = .11$, $p = .901$, partial $\eta^2 = .002$.

Personal Meaning Profile

We conducted a multivariate ANOVA with condition (attraction vs. repel vs. non-magnetic) as a fixed factor and the seven subscales of the short Personal Meaning Profile (McDonald, Wong, & Gingras, 2013) entered as dependent variables. Results indicated no effect of condition on six of the seven subscales, as follows:

Achievement Subscale: $F(2, 117) = 1.69$, $p = .190$, partial $\eta^2 = .03$
Relationship Subscale: $F(2, 117) = 1.09$, $p = .340$, partial $\eta^2 = .02$
Self-Transcendence Subscale: $F(2, 117) = .99$, $p = .377$, partial $\eta^2 = .02$
Self-Acceptance Subscale: $F(2, 117) = 1.45$, $p = .239$, partial $\eta^2 = .02$
Intimacy Subscale: $F(2, 117) = 2.04$, $p = .134$, partial $\eta^2 = .03$
Fair Treatment Subscale: $F(2, 117) = 1.18$, $p = .311$, partial $\eta^2 = .02$

A significant effect of condition was observed for the Religion subscale, $F(2, 117) = 3.53$, $p = .032$, partial $\eta^2 = .06$. Bonferroni-adjusted post-hoc tests indicated that participants in the repel condition ($M = 5.32$) endorsed religion as a source of meaning in their lives significantly less than participants in the control condition ($M = 5.81$), $p = .030$. Endorsement of the Religion subscale in the attraction condition ($M = 5.64$) did not differ from either the repel condition, $p = .252$, or from the control condition, $p = 1.00$. This difference was unexpected, and we believe it is likely spurious; there is no immediately clear explanation as to why this manipulation should have influenced participants' reliance on religion as a source of meaning.

Experiences in Close Relationships Scale
We conducted a multivariate ANOVA with condition (attraction vs. repel vs. non-magnetic) as a fixed factor and the Anxiety and Avoidance subscales of the short Experiences in Close Relationships scale (Wei, Russell, Mallinckrodt, & Vogel, 2007) entered as dependent variables. Results indicated no effect of condition on either the Anxiety subscale, $F(2, 117) = .65$, $p = .523$, partial $\eta^2 = .01$, or on the Avoidance subscale, $F(2, 117) = 1.18$, $p = .312$, partial $\eta^2 = .02$.

Study 2
Mood
An independent-samples *t*-test was conducted on the positive and negative affect subscales of the mood measure, which was identical to the measure used in Study 1 (Tsai, 2007). Positive affect did not significantly differ between the attraction condition ($M = 4.70$) and the control condition ($M = 4.45$), $t(148) = 1.50$, $p = .136$. Negative affect was also not found to differ between the attraction condition ($M = 2.54$) and the control conditions ($M = 2.78$), $t(148) = 1.45$, $p = .150$.

Endorsement of Journey and Force Metaphors for Romantic Love
Participants completed an ad hoc measure of subjective endorsement of journey metaphors (11 items, e.g. "You know you're in love when the relationship is on track.") and physical-force metaphors (13 items, e.g. "You know you're in love when there is magnetism between you and someone.") for romantic love. Responses were made on a X-point scale, and the items from each subscale were averaged to yield the final scores for endorsement of journey ($M = 4.54$, $SD = 1.10$, $\alpha = .89$) and force metaphors ($M = 4.35$, $SD = 1.15$, $\alpha = .92$).

An independent-samples *t*-test was conducted to examine whether the experimental manipulation affected explicit endorsement of these metaphors for romantic love. Endorsement of journey metaphors did not differ between the attraction ($M = 4.53$) and control conditions ($M = 4.54$), $t(148) = .01$, $p = .989$. Endorsement of force metaphors also did not differ between the attraction ($M = 4.34$) and control conditions ($M = 4.36$), $t(148) = .07$, $p = .944$. These results clearly show that the manipulation did not affect participants' explicit agreement with metaphoric statements concerning love.

PROCESS Output: Mediation of Effects by Accessibility of Romantic Thoughts
Model 1: DV = Relationship Satisfaction

```
Run MATRIX procedure:

************** PROCESS Procedure for SPSS Release 2.15 ******************
```

```
          Written by Andrew F. Hayes, Ph.D.       www.afhayes.com
    Documentation available in Hayes (2013). www.guilford.com/p/hayes3

**************************************************************************
Model = 4
    Y = sat
    X = Cond
    M = ACCESS_T

Sample size
        150

**************************************************************************
Outcome: ACCESS_T

Model Summary
          R       R-sq        MSE          F        df1        df2          p
      .1750      .0306      .8216     4.6737     1.0000   148.0000      .0322

Model
              coeff         se          t          p       LLCI       ULCI
constant     2.0000      .2340     8.5455      .0000     1.5375     2.4625
Cond         -.3200      .1480    -2.1619      .0322     -.6125     -.0275

**************************************************************************
Outcome: sat

Model Summary
          R       R-sq        MSE          F        df1        df2          p
      .2154      .0464     1.8169     3.5755     2.0000   147.0000      .0305

Model
              coeff         se          t          p       LLCI       ULCI
constant     5.3396      .4253    12.5545      .0000     4.4991     6.1801
ACCESS_T      .2049      .1222     1.6760      .0959     -.0367      .4464
Cond         -.3931      .2236    -1.7584      .0808     -.8349      .0487

******************** DIRECT AND INDIRECT EFFECTS *************************

Direct effect of X on Y
     Effect         SE          t          p       LLCI       ULCI
     -.3931      .2236    -1.7584      .0808     -.8349      .0487

Indirect effect of X on Y
             Effect    Boot SE   BootLLCI   BootULCI
ACCESS_T     -.0656      .0489     -.2158     -.0019

Partially standardized indirect effect of X on Y
             Effect    Boot SE   BootLLCI   BootULCI
ACCESS_T     -.0478      .0356     -.1558     -.0008

Completely standardized indirect effect of X on Y
             Effect    Boot SE   BootLLCI   BootULCI
ACCESS_T     -.0240      .0178     -.0781     -.0006

Ratio of indirect to total effect of X on Y
             Effect    Boot SE   BootLLCI   BootULCI
```

```
ACCESS_T      .1429    32.1420     -.0132     1.8667

Ratio of indirect to direct effect of X on Y
            Effect    Boot SE   BootLLCI   BootULCI
ACCESS_T      .1668    20.3605     -.0220     3.8392

R-squared mediation effect size (R-sq_med)
            Effect    Boot SE   BootLLCI   BootULCI
ACCESS_T      .0081      .0075      .0002      .0384

Preacher and Kelley (2011) Kappa-squared
            Effect    Boot SE   BootLLCI   BootULCI
ACCESS_T      .0240      .0167      .0027      .0753

******************* ANALYSIS NOTES AND WARNINGS ************************

Number of bootstrap samples for bias corrected bootstrap confidence
intervals:
     5000

Level of confidence for all confidence intervals in output:
    95.00

------ END MATRIX -----
```

<u>Model 2: DV = Attraction</u>

```
Run MATRIX procedure:

************** PROCESS Procedure for SPSS Release 2.15 ******************

          Written by Andrew F. Hayes, Ph.D.       www.afhayes.com
    Documentation available in Hayes (2013). www.guilford.com/p/hayes3

**************************************************************************
Model = 4
    Y = attract
    X = Cond
    M = ACCESS_T

Sample size
        150

**************************************************************************
Outcome: ACCESS_T

Model Summary
          R       R-sq        MSE          F        df1        df2          p
      .1750      .0306      .8216     4.6737     1.0000   148.0000      .0322

Model
              coeff         se          t          p       LLCI       ULCI
constant     2.0000      .2340     8.5455      .0000     1.5375     2.4625
Cond         -.3200      .1480    -2.1619      .0322     -.6125     -.0275

**************************************************************************
```

```
Outcome: attract

Model Summary
          R       R-sq        MSE          F        df1        df2          p
      .2730      .0745     1.6706     5.9186     2.0000   147.0000      .0034

Model
              coeff         se          t          p       LLCI       ULCI
constant     5.8451      .4078    14.3322      .0000     5.0392     6.6511
ACCESS_T      .2228      .1172     1.9005      .0593     -.0089      .4544
Cond         -.5340      .2144    -2.4912      .0138     -.9577     -.1104

******************** DIRECT AND INDIRECT EFFECTS *************************

Direct effect of X on Y
     Effect         SE          t          p       LLCI       ULCI
     -.5340      .2144    -2.4912      .0138     -.9577     -.1104

Indirect effect of X on Y
             Effect    Boot SE   BootLLCI   BootULCI
ACCESS_T     -.0713      .0535     -.2275     -.0024

Partially standardized indirect effect of X on Y
             Effect    Boot SE   BootLLCI   BootULCI
ACCESS_T     -.0534      .0396     -.1638     -.0011

Completely standardized indirect effect of X on Y
             Effect    Boot SE   BootLLCI   BootULCI
ACCESS_T     -.0268      .0198     -.0818     -.0006

Ratio of indirect to total effect of X on Y
             Effect    Boot SE   BootLLCI   BootULCI
ACCESS_T      .1178      .3346     -.0021      .5924

Ratio of indirect to direct effect of X on Y
             Effect    Boot SE   BootLLCI   BootULCI
ACCESS_T      .1335     7.3062     -.0058     1.1675

R-squared mediation effect size (R-sq_med)
             Effect    Boot SE   BootLLCI   BootULCI
ACCESS_T      .0127      .0100      .0010      .0458

Preacher and Kelley (2011) Kappa-squared
             Effect    Boot SE   BootLLCI   BootULCI
ACCESS_T      .0271      .0190      .0029      .0813

******************** ANALYSIS NOTES AND WARNINGS *************************

Number of bootstrap samples for bias corrected bootstrap confidence
intervals:
     5000

Level of confidence for all confidence intervals in output:
    95.00

------ END MATRIX -----
```

Model 3: DV = Intimacy

```
Run MATRIX procedure:

************** PROCESS Procedure for SPSS Release 2.15 *******************

          Written by Andrew F. Hayes, Ph.D.       www.afhayes.com
    Documentation available in Hayes (2013). www.guilford.com/p/hayes3

**************************************************************************
Model = 4
    Y = intimacy
    X = Cond
    M = ACCESS_T

Sample size
        150

**************************************************************************
Outcome: ACCESS_T

Model Summary
          R       R-sq        MSE          F        df1        df2          p
      .1750      .0306      .8216     4.6737     1.0000   148.0000      .0322

Model
              coeff         se          t          p       LLCI       ULCI
constant     2.0000      .2340     8.5455      .0000     1.5375     2.4625
Cond         -.3200      .1480    -2.1619      .0322     -.6125     -.0275

**************************************************************************
Outcome: intimacy

Model Summary
          R       R-sq        MSE          F        df1        df2          p
      .1740      .0303     1.7189     2.2943     2.0000   147.0000      .1044

Model
              coeff         se          t          p       LLCI       ULCI
constant     6.1631      .4137    14.8981      .0000     5.3455     6.9806
ACCESS_T      .0545      .1189      .4582      .6475     -.1805      .2894
Cond         -.4306      .2174    -1.9801      .0496     -.8603     -.0008

******************** DIRECT AND INDIRECT EFFECTS *************************

Direct effect of X on Y
     Effect         SE          t          p       LLCI       ULCI
     -.4306      .2174    -1.9801      .0496     -.8603     -.0008

Indirect effect of X on Y
             Effect    Boot SE   BootLLCI   BootULCI
ACCESS_T     -.0174      .0434     -.1234      .0612

Partially standardized indirect effect of X on Y
             Effect    Boot SE   BootLLCI   BootULCI
```

```
ACCESS_T     -.0132      .0330     -.0938      .0469

Completely standardized indirect effect of X on Y
             Effect    Boot SE   BootLLCI   BootULCI
ACCESS_T     -.0066      .0165     -.0470      .0234

Ratio of indirect to total effect of X on Y
             Effect    Boot SE   BootLLCI   BootULCI
ACCESS_T      .0389 6.414E+011     -.2067      .5861

Ratio of indirect to direct effect of X on Y
             Effect    Boot SE   BootLLCI   BootULCI
ACCESS_T      .0405      .8926     -.1816      .8573

R-squared mediation effect size (R-sq_med)
             Effect    Boot SE   BootLLCI   BootULCI
ACCESS_T      .0030      .0065     -.0042      .0270

Preacher and Kelley (2011) Kappa-squared
             Effect    Boot SE   BootLLCI   BootULCI
ACCESS_T      .0066      .0118      .0000      .0289

******************** ANALYSIS NOTES AND WARNINGS *************************

Number of bootstrap samples for bias corrected bootstrap confidence
intervals:
     5000

Level of confidence for all confidence intervals in output:
    95.00

------ END MATRIX -----
```

<u>Model 4: DV = Commitment</u>

```
Run MATRIX procedure:

************** PROCESS Procedure for SPSS Release 2.15 *******************

          Written by Andrew F. Hayes, Ph.D.       www.afhayes.com
    Documentation available in Hayes (2013). www.guilford.com/p/hayes3

**************************************************************************
Model = 4
    Y = commit
    X = Cond
    M = ACCESS_T

Sample size
        150

**************************************************************************
Outcome: ACCESS_T

Model Summary
          R       R-sq        MSE          F        df1        df2          p
```

```
      .1750      .0306      .8216     4.6737     1.0000   148.0000      .0322

Model
              coeff         se          t          p       LLCI       ULCI
constant     2.0000      .2340     8.5455      .0000     1.5375     2.4625
Cond         -.3200      .1480    -2.1619      .0322     -.6125     -.0275

**************************************************************************
Outcome: commit

Model Summary
          R       R-sq        MSE          F        df1        df2          p
      .2186      .0478     2.2927     3.6882     2.0000   147.0000      .0274

Model
              coeff         se          t          p       LLCI       ULCI
constant     5.1640      .4778    10.8084      .0000     4.2198     6.1082
ACCESS_T      .2751      .1373     2.0038      .0469      .0038      .5465
Cond         -.3653      .2511    -1.4545      .1479     -.8616      .1310

******************** DIRECT AND INDIRECT EFFECTS *************************

Direct effect of X on Y
     Effect         SE          t          p       LLCI       ULCI
     -.3653      .2511    -1.4545      .1479     -.8616      .1310

Indirect effect of X on Y
             Effect    Boot SE   BootLLCI   BootULCI
ACCESS_T     -.0880      .0659     -.2739     -.0027

Partially standardized indirect effect of X on Y
             Effect    Boot SE   BootLLCI   BootULCI
ACCESS_T     -.0571      .0419     -.1751     -.0018

Completely standardized indirect effect of X on Y
             Effect    Boot SE   BootLLCI   BootULCI
ACCESS_T     -.0287      .0210     -.0875     -.0010

Ratio of indirect to total effect of X on Y
             Effect    Boot SE   BootLLCI   BootULCI
ACCESS_T      .1942     3.5677     -.0334     2.7836

Ratio of indirect to direct effect of X on Y
             Effect    Boot SE   BootLLCI   BootULCI
ACCESS_T      .2410     6.7272     -.1246    11.8674

R-squared mediation effect size (R-sq_med)
             Effect    Boot SE   BootLLCI   BootULCI
ACCESS_T      .0081      .0084     -.0001      .0399

Preacher and Kelley (2011) Kappa-squared
             Effect    Boot SE   BootLLCI   BootULCI
ACCESS_T      .0285      .0200      .0031      .0853

******************** ANALYSIS NOTES AND WARNINGS *************************
```

```
Number of bootstrap samples for bias corrected bootstrap confidence
intervals:
     5000

Level of confidence for all confidence intervals in output:
    95.00

------ END MATRIX -----
```

PROCESS Output: Alternative Models Testing Mediation of Effects on Romantic-Thought Accessibility Through Relationship Satisfaction, Attraction, Intimacy, and Commitment

Model 1: Simultaneous Mediation by All Four Variables

```
Run MATRIX procedure:

************** PROCESS Procedure for SPSS Release 2.15 ******************

          Written by Andrew F. Hayes, Ph.D.       www.afhayes.com
    Documentation available in Hayes (2013). www.guilford.com/p/hayes3

**************************************************************************
Model = 4
    Y = ACCESS_T
    X = Cond
   M1 = sat
   M2 = attract
   M3 = intimacy
   M4 = commit

Sample size
        150

**************************************************************************
Outcome: sat

Model Summary
          R       R-sq        MSE          F        df1        df2          p
      .1678      .0282     1.8391     4.2896     1.0000   148.0000      .0401

Model
              coeff         se          t          p       LLCI       ULCI
constant     5.7493      .3502    16.4195      .0000     5.0574     6.4413
Cond         -.4587      .2215    -2.0711      .0401     -.8963     -.0210

**************************************************************************
Outcome: attract

Model Summary
          R       R-sq        MSE          F        df1        df2          p
      .2276      .0518     1.7001     8.0825     1.0000   148.0000      .0051

Model
              coeff         se          t          p       LLCI       ULCI
constant     6.2907      .3367    18.6855      .0000     5.6254     6.9559
Cond         -.6053      .2129    -2.8430      .0051    -1.0261     -.1846
```

```
**************************************************************************
Outcome: intimacy

Model Summary
          R       R-sq        MSE          F        df1        df2          p
      .1700      .0289     1.7097     4.4022     1.0000   148.0000      .0376

Model
              coeff         se          t          p       LLCI       ULCI
constant     6.2720      .3376    18.5778      .0000     5.6048     6.9392
Cond         -.4480      .2135    -2.0981      .0376     -.8699     -.0261

**************************************************************************
Outcome: commit

Model Summary
          R       R-sq        MSE          F        df1        df2          p
      .1476      .0218     2.3395     3.2942     1.0000   148.0000      .0715

Model
              coeff         se          t          p       LLCI       ULCI
constant     5.7143      .3949    14.4694      .0000     4.9339     6.4947
Cond         -.4533      .2498    -1.8150      .0715     -.9469      .0402

**************************************************************************
Outcome: ACCESS_T

Model Summary
          R       R-sq        MSE          F        df1        df2          p
      .2709      .0734      .8072     2.2806     5.0000   144.0000      .0497

Model
              coeff         se          t          p       LLCI       ULCI
constant     1.5144      .4767     3.1772      .0018      .5723     2.4566
sat           .0794      .0836      .9497      .3438     -.0859      .2447
attract       .0712      .1118      .6362      .5256     -.1499      .2922
intimacy     -.1299      .0843    -1.5408      .1256     -.2965      .0367
commit        .0693      .1042      .6650      .5071     -.1367      .2753
Cond         -.2673      .1519    -1.7594      .0806     -.5675      .0330

******************** DIRECT AND INDIRECT EFFECTS *************************

Direct effect of X on Y
     Effect         SE          t          p       LLCI       ULCI
     -.2673      .1519    -1.7594      .0806     -.5675      .0330

Indirect effect of X on Y
             Effect    Boot SE   BootLLCI   BootULCI
TOTAL        -.0527      .0515     -.1590      .0441
sat          -.0364      .0489     -.1745      .0296
attract      -.0431      .0719     -.2207      .0786
intimacy      .0582      .0551     -.0100      .2193
commit       -.0314      .0554     -.1917      .0438

Partially standardized indirect effect of X on Y
             Effect    Boot SE   BootLLCI   BootULCI
TOTAL        -.0575      .0551     -.1684      .0500
```

```
sat           -.0397      .0530     -.1861      .0329
attract       -.0469      .0779     -.2312      .0887
intimacy       .0634      .0599     -.0108      .2393
commit        -.0342      .0606     -.2132      .0480

Completely standardized indirect effect of X on Y
             Effect    Boot SE   BootLLCI   BootULCI
TOTAL         -.0288      .0275     -.0847      .0245
sat           -.0199      .0265     -.0932      .0163
attract       -.0236      .0390     -.1160      .0440
intimacy       .0318      .0300     -.0054      .1200
commit        -.0172      .0303     -.1067      .0239

Ratio of indirect to total effect of X on Y
             Effect    Boot SE   BootLLCI   BootULCI
TOTAL          .1648 6.656E+011     -.2425      .9988
sat            .1138 4.273E+010     -.1292     1.0627
attract        .1346 2.076E+010     -.3495     1.6656
intimacy      -.1819 3.410E+011    -1.4228      .0722
commit         .0982 3.467E+011     -.1952     1.0769

Ratio of indirect to direct effect of X on Y
             Effect    Boot SE   BootLLCI   BootULCI
TOTAL          .1973    14.9188     -.5309     3.0611
sat            .1363     4.2933     -.2005     2.9548
attract        .1612    14.8803     -.4518     4.3813
intimacy      -.2177     2.4030    -3.0689      .1530
commit         .1175     3.4141     -.3068     1.9759

******************** ANALYSIS NOTES AND WARNINGS *************************

Number of bootstrap samples for bias corrected bootstrap confidence
intervals:
     5000

Level of confidence for all confidence intervals in output:
    95.00

------ END MATRIX -----
```

Model 2: DV = Romantic-Thought Accessibility, Mediator = Relationship Satisfaction

```
Run MATRIX procedure:

************** PROCESS Procedure for SPSS Release 2.15 *******************

          Written by Andrew F. Hayes, Ph.D.       www.afhayes.com
    Documentation available in Hayes (2013). www.guilford.com/p/hayes3

**************************************************************************
Model = 4
    Y = ACCESS_T
    X = Cond
    M = sat

Sample size
        150
```

```
**************************************************************************
Outcome: sat

Model Summary
          R       R-sq        MSE          F        df1        df2          p
      .1678      .0282     1.8391     4.2896     1.0000   148.0000      .0401

Model
              coeff         se          t          p       LLCI       ULCI
constant     5.7493      .3502    16.4195      .0000     5.0574     6.4413
Cond         -.4587      .2215    -2.0711      .0401     -.8963     -.0210

**************************************************************************
Outcome: ACCESS_T

Model Summary
          R       R-sq        MSE          F        df1        df2          p
      .2209      .0488      .8117     3.7699     2.0000   147.0000      .0253

Model
              coeff         se          t          p       LLCI       ULCI
constant     1.4738      .3908     3.7717      .0002      .7016     2.2460
sat           .0915      .0546     1.6760      .0959     -.0164      .1994
Cond         -.2780      .1492    -1.8629      .0645     -.5730      .0169

******************** DIRECT AND INDIRECT EFFECTS *************************

Direct effect of X on Y
     Effect         SE          t          p       LLCI       ULCI
     -.2780      .1492    -1.8629      .0645     -.5730      .0169

Indirect effect of X on Y
        Effect    Boot SE   BootLLCI   BootULCI
sat     -.0420      .0337     -.1387      .0014

Partially standardized indirect effect of X on Y
        Effect    Boot SE   BootLLCI   BootULCI
sat     -.0458      .0363     -.1479      .0020

Completely standardized indirect effect of X on Y
        Effect    Boot SE   BootLLCI   BootULCI
sat     -.0230      .0181     -.0745      .0008

Ratio of indirect to total effect of X on Y
        Effect    Boot SE   BootLLCI   BootULCI
sat      .1312 2.436E+010     -.0172     1.6283

Ratio of indirect to direct effect of X on Y
        Effect    Boot SE   BootLLCI   BootULCI
sat      .1510     2.0151     -.0276     3.2106

R-squared mediation effect size (R-sq_med)
        Effect    Boot SE   BootLLCI   BootULCI
sat      .0082      .0079      .0001      .0370

Preacher and Kelley (2011) Kappa-squared
        Effect    Boot SE   BootLLCI   BootULCI
```

```
sat        .0230       .0171       .0016       .0719

******************** ANALYSIS NOTES AND WARNINGS *************************

Number of bootstrap samples for bias corrected bootstrap confidence
intervals:
     5000

Level of confidence for all confidence intervals in output:
    95.00

------ END MATRIX -----
```

Model 3: DV = Romantic-Thought Accessibility, Mediator = Attraction

```
Run MATRIX procedure:

************** PROCESS Procedure for SPSS Release 2.15 *******************

          Written by Andrew F. Hayes, Ph.D.       www.afhayes.com
    Documentation available in Hayes (2013). www.guilford.com/p/hayes3

**************************************************************************
Model = 4
    Y = ACCESS_T
    X = Cond
    M = attract

Sample size
        150

**************************************************************************
Outcome: attract

Model Summary
          R       R-sq        MSE          F        df1        df2          p
      .2276      .0518     1.7001     8.0825     1.0000   148.0000      .0051

Model
              coeff         se          t          p       LLCI       ULCI
constant     6.2907      .3367    18.6855      .0000     5.6254     6.9559
Cond         -.6053      .2129    -2.8430      .0051    -1.0261     -.1846

**************************************************************************
Outcome: ACCESS_T

Model Summary
          R       R-sq        MSE          F        df1        df2          p
      .2321      .0539      .8074     4.1841     2.0000   147.0000      .0171

Model
              coeff         se          t          p       LLCI       ULCI
constant     1.3228      .4252     3.1108      .0022      .4824     2.1631
attract       .1077      .0566     1.9005      .0593     -.0043      .2196
Cond         -.2548      .1507    -1.6912      .0929     -.5526      .0430

******************** DIRECT AND INDIRECT EFFECTS *************************
```

```
Direct effect of X on Y
     Effect         SE          t          p       LLCI       ULCI
     -.2548      .1507    -1.6912      .0929     -.5526      .0430

Indirect effect of X on Y
             Effect    Boot SE   BootLLCI   BootULCI
attract      -.0652      .0403     -.1721     -.0057

Partially standardized indirect effect of X on Y
             Effect    Boot SE   BootLLCI   BootULCI
attract      -.0710      .0427     -.1799     -.0065

Completely standardized indirect effect of X on Y
             Effect    Boot SE   BootLLCI   BootULCI
attract      -.0356      .0213     -.0905     -.0033

Ratio of indirect to total effect of X on Y
             Effect    Boot SE   BootLLCI   BootULCI
attract       .2037 4.752E+012     -.0007     1.4975

Ratio of indirect to direct effect of X on Y
             Effect    Boot SE   BootLLCI   BootULCI
attract       .2557     7.5752     -.0343     3.5950

R-squared mediation effect size (R-sq_med)
             Effect    Boot SE   BootLLCI   BootULCI
attract       .0122      .0103      .0006      .0465

Preacher and Kelley (2011) Kappa-squared
             Effect    Boot SE   BootLLCI   BootULCI
attract       .0352      .0202      .0047      .0875

******************** ANALYSIS NOTES AND WARNINGS *************************

Number of bootstrap samples for bias corrected bootstrap confidence
intervals:
     5000

Level of confidence for all confidence intervals in output:
    95.00

------ END MATRIX -----
```

<u>Model 4: DV = Romantic-Thought Accessibility, Mediator = Intimacy</u>

```
Run MATRIX procedure:

************** PROCESS Procedure for SPSS Release 2.15 *******************

          Written by Andrew F. Hayes, Ph.D.       www.afhayes.com
    Documentation available in Hayes (2013). www.guilford.com/p/hayes3

**************************************************************************
Model = 4
    Y = ACCESS_T
    X = Cond
```

```
    M = intimacy

Sample size
        150

**************************************************************************
Outcome: intimacy

Model Summary
          R       R-sq        MSE          F        df1        df2          p
      .1700      .0289     1.7097     4.4022     1.0000   148.0000      .0376

Model
              coeff         se          t          p       LLCI       ULCI
constant     6.2720      .3376    18.5778      .0000     5.6048     6.9392
Cond         -.4480      .2135    -2.0981      .0376     -.8699     -.0261

**************************************************************************
Outcome: ACCESS_T

Model Summary
          R       R-sq        MSE          F        df1        df2          p
      .1789      .0320      .8260     2.4293     2.0000   147.0000      .0916

Model
              coeff         se          t          p       LLCI       ULCI
constant     1.8358      .4284     4.2857      .0000      .9893     2.6823
intimacy      .0262      .0571      .4582      .6475     -.0867      .1391
Cond         -.3083      .1506    -2.0469      .0425     -.6059     -.0106

******************** DIRECT AND INDIRECT EFFECTS *************************

Direct effect of X on Y
     Effect         SE          t          p       LLCI       ULCI
     -.3083      .1506    -2.0469      .0425     -.6059     -.0106

Indirect effect of X on Y
             Effect    Boot SE   BootLLCI   BootULCI
intimacy     -.0117      .0313     -.0958      .0361

Partially standardized indirect effect of X on Y
             Effect    Boot SE   BootLLCI   BootULCI
intimacy     -.0128      .0342     -.1043      .0403

Completely standardized indirect effect of X on Y
             Effect    Boot SE   BootLLCI   BootULCI
intimacy     -.0064      .0171     -.0523      .0202

Ratio of indirect to total effect of X on Y
             Effect    Boot SE   BootLLCI   BootULCI
intimacy      .0366 3.071E+012     -.1458      .7360

Ratio of indirect to direct effect of X on Y
             Effect    Boot SE   BootLLCI   BootULCI
intimacy      .0380     5.7937     -.1396     1.3222

R-squared mediation effect size (R-sq_med)
```

```
             Effect    Boot SE   BootLLCI   BootULCI
intimacy      .0030       .0064     -.0041      .0253

Preacher and Kelley (2011) Kappa-squared
             Effect    Boot SE   BootLLCI   BootULCI
intimacy      .0065       .0129      .0000      .0318

******************* ANALYSIS NOTES AND WARNINGS ************************

Number of bootstrap samples for bias corrected bootstrap confidence
intervals:
     5000

Level of confidence for all confidence intervals in output:
    95.00

------ END MATRIX -----
```

<u>Model 5: DV = Romantic-Thought Accessibility, Mediator = Commitment</u>

```
  Run MATRIX procedure:

************** PROCESS Procedure for SPSS Release 2.15 ******************

          Written by Andrew F. Hayes, Ph.D.       www.afhayes.com
    Documentation available in Hayes (2013). www.guilford.com/p/hayes3

**************************************************************************
Model = 4
    Y = ACCESS_T
    X = Cond
    M = commit

Sample size
        150

**************************************************************************
Outcome: commit

Model Summary
          R       R-sq        MSE          F        df1        df2          p
      .1476      .0218     2.3395     3.2942     1.0000   148.0000      .0715

Model
              coeff         se          t          p       LLCI       ULCI
constant     5.7143      .3949    14.4694      .0000     4.9339     6.4947
Cond         -.4533      .2498    -1.8150      .0715     -.9469      .0402

**************************************************************************
Outcome: ACCESS_T

Model Summary
          R       R-sq        MSE          F        df1        df2          p
      .2375      .0564      .8052     4.3920     2.0000   147.0000      .0140

Model
              coeff         se          t          p       LLCI       ULCI
```

```
constant     1.4478      .3600     4.0214      .0001      .7363     2.1593
commit        .0966      .0482     2.0038      .0469      .0013      .1919
Cond         -.2762      .1482    -1.8642      .0643     -.5690      .0166

******************** DIRECT AND INDIRECT EFFECTS *************************

Direct effect of X on Y
     Effect         SE          t          p       LLCI       ULCI
     -.2762      .1482    -1.8642      .0643     -.5690      .0166

Indirect effect of X on Y
           Effect    Boot SE   BootLLCI   BootULCI
commit     -.0438      .0346     -.1410      .0015

Partially standardized indirect effect of X on Y
           Effect    Boot SE   BootLLCI   BootULCI
commit     -.0477      .0374     -.1534      .0018

Completely standardized indirect effect of X on Y
           Effect    Boot SE   BootLLCI   BootULCI
commit     -.0240      .0187     -.0769      .0009

Ratio of indirect to total effect of X on Y
           Effect    Boot SE   BootLLCI   BootULCI
commit      .1369     1.4921     -.0177     1.1560

Ratio of indirect to direct effect of X on Y
           Effect    Boot SE   BootLLCI   BootULCI
commit      .1586     2.9988     -.0252     3.1010

R-squared mediation effect size (R-sq_med)
           Effect    Boot SE   BootLLCI   BootULCI
commit      .0083      .0085     -.0001      .0390

Preacher and Kelley (2011) Kappa-squared
           Effect    Boot SE   BootLLCI   BootULCI
commit      .0241      .0177      .0022      .0753

******************** ANALYSIS NOTES AND WARNINGS *************************

Number of bootstrap samples for bias corrected bootstrap confidence
intervals:
     5000

Level of confidence for all confidence intervals in output:
    95.00

------ END MATRIX -----
```
